# A cross-sectional questionnaire survey on knowledge of anti-protozoal drug use and resistance among AHPs in Kwara State, Nigeria

**DOI:** 10.1186/s12917-022-03331-3

**Published:** 2022-06-07

**Authors:** Nusirat Elelu, Grace Agene, Fatima Sanusi, Ahmad Ibrahim Al-Mustapha

**Affiliations:** 1 Department of Veterinary Public Health and Preventive Medicine, Faculty of Veterinary Medicine, University of Ilorin, Ilorin, Kwara State Nigeria; 2 Kwara State Primary Healthcare Development Agency, Ilorin, Kwara State Nigeria; 3 Department of Veterinary Biochemistry and Physiology, Faculty of Veterinary Medicine, University of Ilorin, Ilorin, Kwara State Nigeria; 4 Department of Veterinary Services, Kwara State Ministry of Agriculture and Rural Development, Ilorin, Kwara State, Nigeria; 5 Department of Veterinary Public Health and Preventive Medicine, Faculty of Veterinary Medicine, University of Ibadan, Ibadan, Oyo State Nigeria; 6 Infectious Disease and One Health, Faculty of Pharmaceutical Sciences, Universite de Tours, Tours, France

**Keywords:** Anti-protozoan resistance, Antimicrobial resistance, AHPs, Kwara, Nigeria

## Abstract

Antimicrobial resistance is a global public health crisis. However, there is a paucity of data on anti-protozoal resistance (APR), especially in animals. Hence, we assessed the knowledge of prudent antiprotozoal drug usage (APU) and resistance among animal health practitioners (AHPs) in Kwara State, Nigeria.

A cross-sectional survey of 435 AHPs was performed in Kwara State from the 5th of March to the 31st of July 2020 using a structured and validated questionnaire. We used logistic regression analysis to identify socio-demographic factors that are associated with the satisfactory perception of prudent APU and good knowledge of APR among AHPs.

Our findings showed that 80.2% (*n* = 349) of the AHPs in Kwara state had a good knowledge of APR with a mean knowledge score of 5.8 ± 1.2. In the same vein, 75.6% (*n* = 329) of the AHPs had a satisfactory perception of prudent APU with a mean score of 3.84 ± 1.21. Only 10.1% of the AHPs had observed treatment failures after the use of anti-protozoal drugs. Most of the AHPs (75.6%) believed that APR poses a significant threat to animal production and health globally. Logistic regression analysis showed that female AHPs were more likely (OR: 2.17; 95% CI: 0.91, 5.20; *p* < 0.005) to have better knowledge of APR than their male counterparts. AHPs with tertiary education were likely (OR: 2.77; 95% CI: 0.96, 4.99; *p* < 0.05) to be more knowledgeable about APR and have satisfactory perceptions of APU (OR: 1.57; 95% CI: 1.16, 2.99; *p* = 0.07) respectively. Finally, veterinarians were 3.76 times (95% CI:1.26, 9.25; *p* < 0.001) more likely to have good knowledge of APR and better perceptions of APU (OR: 3.28; 95% CI: 1.89, 5.68; *p* < 0.001) than other AHPs respectively.

To control antimicrobial resistance, continuous training of AHPs especially para-veterinary officers is essential to update their knowledge on prudent antimicrobial usage and prevent the emergence of resistant protozoan parasites.

**Supplementary Information:**

The online version contains supplementary material available at 10.1186/s12917-022-03331-3.

## Introduction

Antimicrobials are agents that kill microorganisms or stop their growth [1]. This group of naturally occurring agents includes antibiotics, antifungals, antivirals, and antiprotozoals [2]. In most Low-and-Middle income countries such as Nigeria, these classes of drugs are available as over-the-counter drugs [3, 4]. Hence, freely available to all animal health care workers, farmers, and the general public. Over the last few decades, the global consumption of anti-protozoal drugs has skyrocketed especially anti-malarial drugs in humans [5]. Similarly, the consumption of other anti-parasitic drugs used in animals such as anticoccidials, anti-trypanosomes, and anti-babesial drugs has been on the rise [6].

However, with the advent of anti-microbial agents, the world recorded a surge in the resistance to these agents [7–11]. It is estimated that by 2050, antimicrobial resistance (AMR) will result in the death of 10 million persons annually if no concerted global actions are not taken [12]. For instance, chloroquine-resistant *Plasmodium falciparum* have been recorded in the late 1950s in the US. In Nigeria, resistance to antiprotozoal drugs in animals has been reported as far back as 1967 [13]. Similarly, Jones and Davies reported resistance against homidium and isometamedium in 1981 in cows [14]. Since then, several other studies have identified resistant protozoan parasites in animals. Simo et al. identified diminazene aceturate resistant trypanosomes in tsetse fly in Cameroon [15] whereas Obi et al. detected multi-drug resistant *Trypanosoma* isolates in South Eastern Nigeria [16] and other countries [17].

Generally, it is believed that the illicit use of veterinary antimicrobials, as well as acaricides in food animals, meant for human consumption has played a key role in the development of AMR in animals which poses a serious threat to public health [18, 19]. In addition, it has an indirect impact on human health via the occurrence of drug residues in animal products [20]. Anti-protozoal drug resistance occurs either via natural selection or due to selection pressure as a result of sub-therapeutic doses of antimicrobials. Hence, drug-resistance genes in these pathogens (bacteria, viruses, parasites, etc.) inactivates drugs and make them ineffective [1]. Resistance to antiprotozoals has been via two main mechanisms: resistance-associated mutations in the protozoan parasite and selective pressure [15]. The selective pressure has been attributed to poor diagnosis (including laboratory confirmation) of most protozoal diseases, improper use of antiprotozoal drugs, poor drug quality, and non-compliance with the standard treatment regimen. In addition, the use of sub-therapeutic doses as a prophylactic treatment in animals has contributed to the development of resistance in these pathogens [20].

In Kwara State (Nigeria), animal health practitioners (AHPs) usually treat most species of domestic animals ranging from equids (horses), ruminants (cattle, sheep, and goats), pigs, small animals (dogs, cats, and rabbits) and poultry. The most available antiprotozoal drugs in Kwara are diminazene aceturate, amprolium, sulpha drugs, and diclazuril amongst others. However, there is a paucity of information on the perception of AHPs about prudent antiprotozoal drug usage (APU) and their knowledge of antiprotozoal drug resistance (APR). Hence, this study assessed the perception of AHPs on prudent APU and their knowledge of APR.

## Material and methods

### Ethical clearance

The ethical approval for the study was obtained from the ethical review committee of the Faculty of Veterinary Medicine, University of Ilorin, Nigeria (reference number: UERC/FVM/2021/011). Finally, written informed consent was sought from the respondents, and participants could decline participation or opt-out at any time.

### Study area

This study was carried out in Kwara State, North Central Nigeria. The state has a landmass of 32,500km^2^ and a human population of 3,599,800 million [21]. The state connects the Northern and Southern parts of Nigeria and is readily accessible to all parts of the country by air, road, and rail transportation. In Kwara state, antiprotozoals are available as over-the-counter drugs and several brands and combinations (in combination with antibiotics and vitamins) are available.

### Questionnaire design

The raw data for this study was obtained from the animal health workers using a semi-structured pre-validated questionnaire. The questionnaire was designed for this study and was validated by two independent academic examiners to ascertain the instruments’ face validity, content, and technical hitches. Furthermore, we assessed the reliability of the survey instrument using the Cronbach Alpha test (with a score of 0.7). Finally, the questionnaire was pre-tested on twenty AHPs before the deployment of the final version for data collection. The results of the pre-test were not included in the final analysis. The questionnaire was designed in the English language.

The questions contained 27 questions that were grouped into three sections: a). Respondents’ demographic information. b) knowledge and perception of antiprotozoal drug prescription and use. This section examined the respondent’s knowledge of antiprotozoal drugs, their indication, the access and affordability of these drugs, and their perceived efficacy. Finally, section C assessed the knowledge of antiprotozoal drug resistance among AHPs (Supplementary file 1).

### Survey methodology

This study was conducted as a cross-sectional survey of AHPs (veterinarians, clinical year veterinary students, food animal producers, and animal health workers) in Kwara State. The survey was available from the 5th of March to the 31st of July 2020. The questionnaire was administered as a one-on-one interview and also via online social media platforms (WhatsApp, Facebook, E-mail, and telegram) to ease administration to other AHPs. Our inclusion criteria were age (18 years and above), location (Kwara State), and occupation (AHPs).

### Study participants

The sample size (study participants) was calculated using the EPI-INFO (version 7.3.1) statistical software. Due to the paucity of information on the knowledge of AHPs in Kwara State, we assumed that 50% of all AHPs will rationally use antiprotozoals and would be aware of APR. Hence using the 50% prevalence rate at 95% CI, we computed that a minimum of 384 respondents was required. To prevent clustering of responses and increase the intra-cluster variability, we administered the survey instrument to AHPs individually.

### Data analysis

The data obtained from this survey were analyzed using Statistical Package for Social Sciences (SPSS) version 2016 (IBM Corp., Armonk, N.Y., USA). Data on knowledge and perception measures of respondents on antiprotozoal drug prescription, usage, and resistance were summarized by descriptive statistics such as frequency and percentage.

To determine if AHPs had a satisfactory perception of prudent antiprotozoal usage (APU), and determine their knowledge of antiprotozoal drug resistance, a numeric scoring system was used. Briefly, we added the scores from the two variables independently. The perception of prudent APU was graded on a 5-item scale whereas the knowledge of APR was scored on a 7-item scale. A correct response attracted a score of 1, while an incorrect response attracted a score of 0. Both variables (the perception of APU and knowledge of APR) were then categorized as adequate (satisfactory) and inadequate (unsatisfactory) using 50% of the maximum obtainable score as the cut-off. Hence, AHPs that had a cumulative score between 4–5 and 4–8 respectively were graded to have an adequate perception of APU and adequate knowledge level on APR whereas those that scored between 0–3 points were graded to have an inadequate perception of APU and inadequate knowledge of APR respectively. We used the Chi-square test to evaluate the association between sociodemographic variables and adequate/inadequate knowledge of antiprotozoal drug resistance at a 95% confidence interval (*p*-value < 0.05 was considered significant). Statistically significant variables were entered into a logistic regression model (univariable and multivariable) to determine the odds ratio at a 95% CI.

## Results

Of the 435 AHPs included in this study, 67.6% (*n* = 294) were male. Almost half (49.2%) of the respondents were aged between 20—29 years. The median age was 28 years with a range of 20 to 61. Veterinarians accounted for 39.5% of all AHPs and 92.6% of the respondents had tertiary education (Table 1).

**Table 1:** Socio-demographic features of AHPs included in this study (*n* = 435)

Demographics	Category	Frequency (%)
Gender	Male	294 (67.6)
	Female	141 (32.4)
Job category	Veterinarian	172 (39.5)
	Clinical Year Veterinary Student	164 (37.7)
	Food Animal Producer	60 (13.8)
	Other Animal Health Worker	39 (9.0)
Age (years)	18 -29	214 (49.2)
	30–39	128 (29.4)
	40–49	57 (13.1)
	> 50	36 (8.3)
Level of Education	Primary school	17 (3.9)
	Secondary school	3 (0.7)
	Tertiary (polytechnics, Universities, etc.)	403 (92.6)
	No formal education	12 (2.8)

### Knowledge of antiprotozoal drug prescription and usage (*n* = 435) among AHPs in Kwara State

**Fig. 1:**
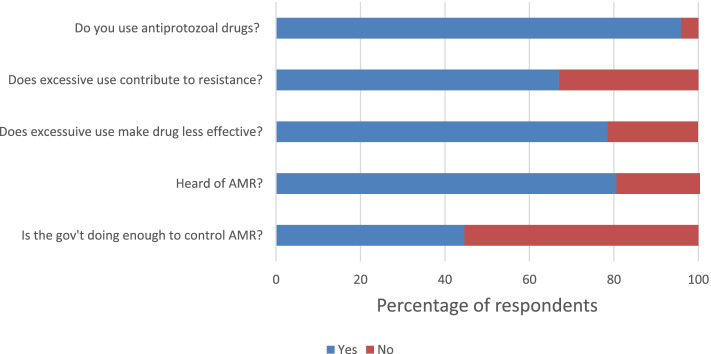
Knowledge of antiprotozoal use and awareness of resistance among AHPs in Kwara State, (*n* = 435)

Of the 435 AHPs included in this survey, most of them (95.9%, *n* = 417) have used antiprotozoal drugs. The most frequently used antiprotozoal drugs among AHPs in Kwara state were: sulpha drugs (87%), diminazene aceturate (59%), amprolium (39%), diclazuril (39%), metronidazole (24%) amongst others that were infrequently used. A large number of respondents (78.6%) agree that unnecessary use of antiprotozoal drugs makes them less effective, while 79.8% know that the use of antiprotozoal without prescription promotes inappropriate use of antiprotozoal drugs (Fig. 1). Furthermore, 83.0% knew that poor clinical diagnosis promotes inappropriate use of antiprotozoal drugs. The majority (78.9%) know that inadequate supervision of antiprotozoal use by qualified health workers promotes inappropriate use of antiprotozoal drugs (Table 2).

**Table 2:** Perception of prudent anti-protozoal usage among AHPs in Kwara State (*n* = 435)

Knowledge of Protozoal Drug Prescription and Use in Animal Health	Frequency (%)
1. Does the use of antiprotozoal without prescription promote inappropriate use of antiprotozoal drugs?	
No	88 (20.2)
Yes	347 (79.8)
2. Does poor clinical diagnosis promote inappropriate use of antiprotozoal drugs?	
No	74 (17)
Yes	361 (83)
3. Does inadequate supervision of antiprotozoal use by qualified health workers promote inappropriate use of antiprotozoal drugs?	
No	92 (21.1)
Yes	343 (78.9)
4. Are there any substitutes for these drugs?	
No	231 (53.1)
Yes	204 (46.9)

Most of the AHPs have used anti-protozoal drugs (APDs) in cows (32.3%, *n* = 235), poultry (33.1%, *n* = 241), and in small animals (18.5%, *n* = 135) than in other animal species. Only 10.1% (*n* = 44) of the AHPs in Kwara State had observed treatment failures and side effects (including mortality) after the use of anti-protozoal drugs. More than half of them (53.3%, *n* = 232) administered anti-protozoal drugs as prophylactic routine medications while 40.7% used anti-protozoal drugs for curative treatments in animals (Table 3). Three-quarter (75.6%, *n* = 329/435) of the AHPs had a satisfactory perception of prudent antiprotozoal prescription and usage.

**Table 3:** Perception of AHPs on antiprotozoal drug prescription and usage (*n* = 435)

Variables	Frequency (%)
1. Has there ever been any observed mortality or side effects after usage of any antiprotozoal drugs on animals?	
No	297 (68.3)
Yes	44 (10.1)
I don’t know	99 (21.4)
2. How available are these drugs?	
Readily available	378 (86.9)
Scarce	20 (4.5)
3. How suitable are these drugs for animal usage?	
Suitable	364 (83.7)
Not suitable	2 (0.5)
Better options	33 (7.6)
4. What purpose(s) have these drugs been administered for?	
Prophylactic	232 (53.3)
Curative	177 (40.7)
Other purposes	23 (5.3)

### Knowledge of anti-protozoal resistance among AHPs in Kwara State

Most of the AHPs (80.2%, *n* = 349) in Kwara state had good knowledge of AMR (APR). Most AHPs obtained information on AMR including APR from clinical experience (24%), literature (53%), and mostly from the internet (61%). The mean knowledge score was 5.8 ± 1.2. Most of the AHPs (75.6%, *n* = 329) also believed that APR poses a significant threat to animal production and health globally. Most AHPs obtained information on APR from the internet (27.5%, *n* = 200). Only 17.2% of the AHPs told their clients (animal owners) of the need to observe the withdrawal period after the use of APDs. More than half of the respondents (64.1%, *n* = 279) believed that educating and re-training AHPs will help reduce the global threat of AMR and APR (Table 4).

**Table 4:** Knowledge and perception of AHPs on antiprotozoal drug resistance (*n* = 435)

Knowledge of APR	Yes (%)	No (%)	I Do not know (%)
1. Have you heard of antiprotozoal drug resistance?	349 (80.2)	86 (19.8)	0
2. Does inappropriate use of antiprotozoal drugs put animals at risk?	368 (84.6)	67 (15.4)	0
3. Do you observe the withdrawal period after the use of anti-protozoal drugs?	75 (17.2)	360 (82.8)	0
4. Does antiprotozoal drug resistance pose a threat to animal health globally?	329 (75.6)	106 (24.4)	0
5. Do you think educating people ignorant on the appropriate antiprotozoal drug use will have a positive effect on decreasing the risk of antiprotozoal drug resistance?	279 (64.1)	75 (17.2)	81 (18.6)

### Impact of socio-demographic factors on knowledge of antiprotozoal drug resistance in Kwara State

There was a positive statistically significant correlation between the socio-demographic variables (gender, age, job category, and educational level) on the knowledge of anti-protozoan resistance (Tables S1; S2). As predicted by the Chi-square analysis, the results of the logistic regression analysis also showed that the four sociodemographic factors (age, gender, job category, and the educational level of the respondent) influenced the knowledge of APR among AHPs in Kwara State. Our findings showed that female AHPs were more likely (OR: 2.17; 95% CI: 0.91, 5.20; *p* < 0.005) to have better knowledge of APR than their male counterparts. AHPs with tertiary education were likely (OR: 2.77; 95% CI: 0.96, 4.99; *p* < 0.05) to be more knowledgeable than others. Finally, veterinarians were 3.76 times more likely to have good knowledge of APR than other AHPs (Table 5).

**Table 5:** Univariable and multivariable logistic regression analysis of demographic variables that affected the knowledge of APR among AHPs in Kwara state

Outcome variable	Variable	Baseline category		OR (95% CI)	*P-value*	OR (95% CI)	*P-value*
		Univariable analysis				Multivariable analysis	
Knowledge of antiprotozoal resistance among AHPs	Age	18–29	30–39	0.47 (0.26, 0.86)	< 0.001	0.36 (0.14, 0.96)	< 0.001
			40–49	0.21 (0.1091, 0.43)		0.98 (0.25, 3.82)	
			> 50	0.17 (0.0805, 0.3885)		0.85 (0.18, 2.87)	
	Gender	Male	Female	1.93 (1.11, 3.36)	< 0.001	2.17 (0.91, 5.20)	< 0.001
	Education	Primary/Secondary	Tertiary	9.58 (6.16, 24.64)	< 0.001	2.77 (0.96, 4.99)	< 0.001
			No formal education	0.55 (0.15, 2.91)		0.18 (0.026, 0.72)	
	Job description	Other Animal Health Worker	Veterinarian	1.12 (0.67, 3.39)	< 0.001	3.76 (1.26, 9.25)	< 0.001
			Clinical year veterinary students	1.39 (0.97, 2.89)		2.37 (0.81, 4.25)	
			Food Animal Producer	0.03 (0.01, 0.08)		0.21 (0.072, 0.635)	

On the other hand, only two of the socio-demographic variables (level of education and job description) significantly influenced the perception of prudent APU among AHPs in Kwara State. AHPs with tertiary education (OR: 1.57; 95% CI: 1.16, 2.99; *p* = 0.07) and those that were veterinarians (OR: 3.28; 95% CI: 1.89, 5.68; *p* < 0.001) were more likely to have satisfactory perception of prudent APU than others (Table 6).

**Table 6:** Univariable and multivariable logistic regression analysis of demographic variables that affected the perception of prudent antiprotozoal usage among AHPs in Kwara state

Outcome variable	Variable	Baseline category		OR (95% CI)	*P-value*	OR (95% CI)	*P-value*
		Univariable analysis				Multivariable analysis	
Perception of prudent antiprotozoal usage among AHPs	Age	18–29	30–39	0.63 (0.38, 1.04)	0.319	-	-
			40–49	0.90 (0.44, 1.81)			
			> 50	0.69 (0.31, 1.54)			
	Gender	Male	Female	1.17 (0.71, 1.84)	0.572	-	-
	Education	Primary/Secondary	Tertiary	1.29 (1.16, 3.01)	0.05	1.57 (1.16, 2.99)	0.07
			No formal education	0.55 (0.15, 1.01)		0.18 (0.04, 0.72)	
	Job description	Other Animal Health Worker	Veterinarian	4.79 (2.89, 9.12)	< 0.001	3.28 (1.89, 5.68)	< 0.001
			Clinical year veterinary students	1.39 (0.72, 2.69)		1.39 (0.74, 2.77)	
			Food Animal Producer	1.48 (0.67, 3.24)		0.29 (0.08, 0.97)	

## Discussion

AMR including antiprotozoal resistance is a public health threat [1]. Controlling AMR must be prioritized to save lives and livelihoods as well as to reduce its economic impact. Two of the five thematic areas of Nigeria’s National Action Plan (NAP) on AMR focused on the creation of awareness among health practitioners (Animal and human health) on prudent antimicrobial usage and Antimicrobial Stewardship programs (AMS) amongst the general public (including health practitioners) [22].

Our findings showed that the majority of the respondents were aware that the illicit and un-prescribed use of antiprotozoal drugs makes them less effective, promotes the emergence of APR pathogens, and has severe economic implications for farmers. This is in agreement with several studies that have reported the lack of restriction on access to antimicrobials especially antiprotozoals as a major contributor to the emergence of AMR pathogens [19, 23–26]. To curtail the emergence and spread of multidrug-resistant (MDR) pathogens in Kwara State, Nigeria and other low-and middle-income countries (LMICs), government agencies should improve their regulatory oversight in monitoring veterinary drugs and restricting access to critical antimicrobials [27]. Furthermore, Elelu N. canvassed for the need to involve more veterinarians in the veterinary drug supply chain and emphasized the proper training and certification of animal health workers in the veterinary pharmaceutical industry to reduce the unprescribed use of antimicrobials [28].

Our findings indicated that although 80% of the AHPs in Kwara state had a good knowledge of APR. This awareness rate is higher than the 49.2% reported by Chukwu et al., among human health workers in Nigeria [29]. In addition, this is higher than the 38% AMR awareness rate and 20% awareness rate reported by Wangmo et al., and Gemeda et al., in Bhutan and Ethiopia respectively [30, 31]. Furthermore, our findings indicated that only 10% of the AHPs in Kwara state had empirically observed treatment failures and side effects (including mortality) after the use of anti-protozoal drugs. This is similar to the report of Gemeda et al., 2020 who reported that only 12% of his respondents in Ethiopia had experienced treatment failures after the use of antimicrobials [31].

The prophylactic use of anti-protozoal drugs by more than half of the AHPs in Kwara State can be attributed to the endemic nature of many protozoan parasites and the lack of any protozoal disease control program. For instance, studies have reported several strains of parasites such as *Babesia* sp., *Theileria* sp., *Trypanosoma* sp., *Toxoplasma* sp., *Eimeria* sp., *Cryptosporidium* sp., etc. which have devastated Nigeria’s animal health sector [32–36]. Hence, AHPs administer prophylactic treatments against these diseases during their peak period. This is particularly more common and important in poultry production where anti-protozoal drugs (anti-coccidiosis or coccidiostats) are used every two weeks in birds raised in the deep-liter system. Furthermore, the lack of ectoparasiticidal control in most extensively raised animals predisposes them to protozoal diseases. This is one of the main indications for the curative use of anti-protozoal drugs in Kwara State.

Several factors could be responsible for the emergence of anti-protozoal-resistant wild-type strains in livestock populations. These include the under-dosing of commonly administered anti-protozoal drugs, the misuse of anti-protozoal drugs, the use of sub-standard and counterfeit drugs, lack of confirmatory diagnosis of diseases, the lack of surveillance programs, and the lack of disease control intervention programs [19, 37].

The perception of AHPs in Kwara State was good as they knew that APR posed a significant threat to animal health globally and that the use of the antimicrobial drug in food-producing animals contributes to the development of resistance in human health. This is similar to the report of Creek and Barrett, 2014 [38]. However, only a few of the AHPs told their clients (animal owners) of the need to observe the withdrawal period after the use of APDs. The lack of observance of withdrawal periods in animals poses the risk of antimicrobial residues to humans through their products such as meat and milk.

To reduce the threat posed by AMR, it is essential to educate and re-train AHPs especially food animal producers and para-veterinarians (other AHPs) on prudent antimicrobial usage, alternatives to antimicrobials (with a focus on the importance of vaccinations, biosecurity, prebiotics, and probiotics) and AMR.

There was a positive statistically significant correlation between the socio-demographic variables of the respondents on their knowledge of anti-protozoan resistance. Our findings showed that female AHPs were more likely to have better knowledge of APR than their male counterparts. This is similar to the report of Wangmo et al., 2020 who also reported better knowledge among female AHPs in Bhutan [30]. Similarly, veterinarians were likely to be more knowledgeable than other AHPs. This is similar to the report of Chukwu et al., who reported in his national survey in Nigeria that medical officers were more knowledgeable than para-medical officers [29]. In addition, the level of education of AHPs greatly influenced their knowledge of AMR and APR. This could be attributed to the fact that highly educated AHPs could have attended training courses on AMR or could have read the literature online about drugs and the development of AMR.

Finally, controlling AMR requires a collaborative, multi-disciplinary approach which must involve awareness creation on AMR, the provision of proper diagnostic and surveillance facilities, and antimicrobial stewardship among AHPs.

Our study has some limitations. The study was conducted in Kwara State; hence the result cannot be representative of other parts of Nigeria. In addition, there is the possibility of misunderstanding the questions by some respondents due to their varying educational levels.

## Conclusion

Protozoan parasites cause significant morbidity, mortality, and economic burden in Nigeria. AHPs have abused anti-protozoal drugs and this has resulted in the emergence of anti-protozoal resistant pathogens. There is the need to increase knowledge of AHPs on prudent use of antiprotozoal drugs and to increase the awareness of APR.

## Supplementary Information

**Additional file 1.** Survey material on “Knowledge and perceptions of AHPs on antiprotozoal drugs usage and resistance in Kwara state”.

**Additional file 2:** **Table S1.** Test of association between socio-demographic variables and the perception of prudent APU among AHPs in Kwara State. **Table S2.** Test of association between socio-demographic variables and the knowledge of APR in Kwara State.

## Data Availability

The dataset generated during the current study will be made available by the corresponding author on reasonable request.
