# Supplementary material for: A cross-sectional questionnaire survey on knowledge of anti-protozoal drug use and resistance among AHPs in Kwara State, Nigeria
Source: BMC Vet Res. 2022 Jun 7;18:214. doi: 10.1186/s12917-022-03331-3 (PMC9172141; doi:10.1186/s12917-022-03331-3)
Supplement: Supplementary file 1 — Additional file 1. Survey material on “Knowledge and perceptions of AHPs on antiprotozoal drugs usage and resistance in Kwara state”. [file 12917_2022_3331_MOESM1_ESM.doc]

**KNOWLEDGE AND PERCEPTIONS OF ANIMAL HEALTH STAKEHOLDERS ON ANTIPROTOZOAL DRUGS PRESCRIPTION AND USAGE: IMPLICATION FOR DEVELOPMENT OF RESISTANCE.**

This study aims to assess the knowledge and perception of animal health practitioners in Kwara State on prudent antimicrobial usage and anti-protozoal resistance. The study is essential to formulate a robust state-wide antimicrobial stewardship program and help improve treatment option in animal health. This will take 3-5 minutes. Your participation is voluntary and you can withdraw at any time. The information will be confidential and for research purposes only. For more information, please contact agenegrace21@gmail.com.

**CONSENT**

Do I have your permission to continue?

Yes (Append signature or thumbprint on consent sheet)

**SECTION A**

**RESPONDENTS’ CHARACTERISTICS**

1. Gender

(a) Male (b) Female

1. Animal Health Job Category

(a) Veterinarian (b) Clinical Year Veterinary Student (c) Food Animal Producer
(d) Other Animal Health Worker

1. Age Category

(a) 18-25 (b) 26 – 35 (c) 36 – 45 (d) Above 45

1. Educational Level

(a) Primary (b) Secondary (c) Tertiary (d) No formal Education

**SECTION B**

**PART 1: KNOWLEDGE AND PERCEPTION ON ANTIPROTOZOAL DRUG PRESCRIPTION AND USE IN ANIMAL HEALTH**

1. Do you use antiprotozoal drugs?

a) Yes (b) No (c) Don’t know

1. What specific disease(s) do you prescribe or use antiprotozoal for?

a) Babesiosis b) Trypanosomiasis c) Coccidiosis d)Others(specify)……………………………

3. Does unnecessary use of antiprotozoal drugs make them less effective?

a) Yes (b) No (c) Don’t know

4. What antiprotozoal drug (s) do you normally use? ……………..…………………………………

5. How available for purchase are these drugs?

a) Readily available b) Scarce c)Not available d) No idea

6. What is your perception towards the suitability of these drugs for animal usage?

a) It is suitable b) It is not suitable c) There are better options
d) I have no idea

7. Does the use of antiprotozoal without prescription promote inappropriate use of antiprotozoal drugs? a) Yes (b) No (c) Don’t know

8. Does poor clinical diagnosis promote inappropriate use of antiprotozoal drugs?

a) Yes (b) No (c) Don’t know

9. Does inadequate supervision of antiprotozoal use by qualified health worker promote inappropriate use of antiprotozoal drugs?

a) Yes (b) No (c) Don’t know

10. What purpose(s)has these drugs being administered for?

A) Prophylactic purpose b) Curative purpose c) Both
d) Others (specify)………………

11. What class(es) of animals are the drugs administered?

a) Cattle b) Small ruminants c) Poultry d) Fish
e) Others (specify)…….............

12. Has there ever been any observed mortality or side effects that you associate with the usage of antiprotozoal drug on animals?

a) Yes (b) No (c) Don’t know

13. If yes, please specify. …………………………………………………
14. Are there any substitutes for these drug agents?

a) Yes(b) No (c) Don’t know

15. If yes, please specify the substitute……………………………………………..

**PART 3: KNOWLEDGE OF ANTIPROTOZOAL DRUG RESISTANCE IN ANIMAL HEALTH**

1. Have you heard about ‘antiprotozoal drug resistance’?

a) Yes (b) No (c) Don’t know

1. Does inappropriate use of antiprotozoal drug put animals at risk?

a) Yes (b) No (c) Don’t know

1. Does misuse of antiprotozoal drug by stakeholders in animal health contributes significantly to development of resistance?

a) Yes (b) No (c) Don’t know

4. Does the frequent use of antiprotozoal drugs lead to resistance thus decrease its efficacy?
 a) Yes (b) No (c) Don’t know

1. Does antiprotozoal drug resistance pose threat to animal health globally?

a) Yes (b) No (c) Don’t know

1. Does the use of antiprotozoal drug in food - producing animal industry (farm animals) contribute to their resistance?

a) Yes (b) No (c) Don’t know

1. Do you think educating lay people on the appropriate antiprotozoal drug use will have a positive effect on decreasing the risk of antiprotozoal drug resistance?

a) Yes (b) No (c) Don’t know

1. Are the Nigerian authorities in charge of animal health doing enough to control misuse of drugs such as antiprotozoal?

a) Yes (b) No (c) Don’t know

1. Respondents’ source(s) of information about antiprotozoal drug resistance

…………………………………………………………
