# Supplementary material for: A cross-sectional questionnaire survey on knowledge of anti-protozoal drug use and resistance among AHPs in Kwara State, Nigeria
Source: BMC Vet Res. 2022 Jun 7;18:214. doi: 10.1186/s12917-022-03331-3 (PMC9172141; doi:10.1186/s12917-022-03331-3)
Supplement: Supplementary file 2 — Additional file 2: Table S1. Test of association between socio-demographic variables and the perception of prudent APU among AHPs in Kwara State. Table S2. Test of association between socio-demographic variables and the knowledge of APR in Kwara State. [file 12917_2022_3331_MOESM2_ESM.docx]

Table S1. Test of association between socio-demographic variables and the perception of prudent APU among AHPs in Kwara State.

| Socio-demographic Feature | Adequate (%) | Inadequate (%) | Total (%) | Chi-square value (ϰ^2^) | P- value |
| --- | --- | --- | --- | --- | --- |
| Gender | | | | | |
| Male | 220 (50.5) | 74 (17) | 294 (67.6) | 0.317 | 0.571 |
| Female | 109 (25) | 32 (7.4) | 141 (32.4) |  |  |
| Job category | | | | | |
| Veterinarian | 114 (26.2) | 58 (13.3) | 172 (39.5) | 19.046 | 0.0001 |
| Clinical Year Vet. Student | 142 (32.6) | 22 (5.1) | 164 (37.7) |  |  |
| Food Animal Producer | 44 (10.1) | 16 (3.7) | 60 (13.8) |  |  |
| Other Animal Health Worker | 29 (6.7) | 10 (2.3) | 39 (9.0) |  |  |
| Age group (years) | | | | | |
| 18-29 | 169 (38.9) | 45 (10.3) | 214 (49.2) | 3.563 | 0.319 |
| 30-39 | 90 (20.7) | 38 (8.7) | 128 (29.4) |  |  |
| 40-49 | 44 (10.1) | 13 (3.0) | 57 (13.1) |  |  |
| >50 | 26 (6) | 10 (2.3) | 36 (8.3) |  |  |
| Educational Level | | | | | |
| Primary school | 10 (3.9) | 0 (3.7) | 10 (2.3) | 10.754 | 0.0001 |
| Secondary school | 17 (3.9) | 2 (0.5) | 19 (4.4) |  |  |
| Tertiary (Polytechnics, Universities, etc.) | 298 (68.5) | 106 (24.4) | 404 (92.9) |  |  |
| No Formal Education | 1 (0.25) | 1 (0.25) | 2 (0.5) |  |  |

^*^ Significant at p<0.05

Table S2. Test of association between socio-demographic variables and the knowledge of APR in Kwara State.

| Socio-demographic Feature | Adequate (%) | Inadequate (%) | Total (%) | Chi-square value (ϰ^2^) | P- value |
| --- | --- | --- | --- | --- | --- |
| Gender | | | | | |
| Male | 204 (46.9) | 90 (20.7) | 294 (67.6) | 7.221 | 0.007 |
| Female | 115 (26.4) | 26 (6.0) | 141 (32.4) |  |  |
| Job category | | | | | |
| Veterinarian | 156 (35.9) | 16 (3.7) | 172 (39.5) | 2.099E2 | 0.0001 |
| Clinical Year Vet. Student | 145 (33.3) | 19 (4.4) | 164 (37.7) |  |  |
| Food Animal Producer | 4 (0.9) | 56 (12.9) | 60 (13.8) |  |  |
| Other Animal Health Worker | 14 (3.2) | 25 (5.7) | 39 (9.0) |  |  |
| Age group (years) | | | | | |
| 18-29 | 174 (40.0) | 40 (9.2) | 214 (49.2) | 25.254 | 0.0001 |
| 30-39 | 95 (21.8) | 33 (7.6) | 128 (29.4) |  |  |
| 40-49 | 31 (7.1) | 26 (6.0) | 57 (13.1) |  |  |
| >50 | 19 (4.4) | 17 (3.9) | 36 (8.3) |  |  |
| Educational Level | | | | | |
| Primary school | 1 (0.2) | 16 (3.7) | 17 (3.9) | 80.853 | 0.0001 |
| Secondary school | 1 (0.2) | 2 (0.5) | 3 (0.7) |  |  |
| Tertiary (Polytechnics, Universities, etc.) | 317 (72.9) | 86 (19.8) | 403 (92.6) |  |  |
| No Formal Education | 0 (0.0) | 12 (2.8) | 12 (2.8) |  |  |

^*^ Significant at p<0.05
